# Higher Education and Reproduction

**Published:** 1915-09

**Authors:** 


**Higher Education and Reproduction.** The Texas Med. Jour., editorial, July, states that 41.9% of the graduates of Mt. Holyoke are married; 32.8% of those of Bryn Mawr; 31% of those of Wellesley. The average reproduction is respectively 0.8%, 1.74, and 1.17 of a child. When we consider that, even under the best conditions, 3 children must be born to a marriage and 4 to the average woman to provide for a stationary population, it is obvious that in spite of contradictions, the highly educated woman is not reproducing her share.

